# Rigidity control mechanism by turgor pressure in plants

**DOI:** 10.1038/s41598-023-29294-5

**Published:** 2023-02-04

**Authors:** Tohya Kanahama, Satoru Tsugawa, Motohiro Sato

**Affiliations:** 1 Graduate School of Engineering, Hokkaido University, Kita 13, Nishi 8, Kita-ku, Sapporo, 060-8628 Japan; 2 Department of Mechanical Engineering, Faculty of Systems Science and Technology, Akita Prefectural University, 84-4 Yurihonjo, Akita, 015-0055 Japan; 3 Faculty of Engineering, Hokkaido University, Kita 13, Nishi 8, Kita-ku, Sapporo, 060-8628 Japan

**Keywords:** Engineering, Physics

## Abstract

The bodies of herbaceous plants are slender, thin, and soft. These plants support their bodies through the action of turgor pressure associated with their internal water stores. The purpose of this study was to apply the principles of structural mechanics to clarify the underlying mechanism of rigidity control that is responsible for turgor pressure in plants and the reason behind the self-supporting ability of herbaceous plants. We modeled a plant a horizontally oriented thin-walled cylindrical cantilever with closed ends enclosing a cavity filled with water that is acted on by its own weight and by internal tension generated through turgor pressure. We derived an equation describing the plant’s consequent deflection, introducing a dimensionless parameter to express the decrease in deflection associated with the action of turgor pressure. We found that the mechanical and physical characteristics of herbaceous plants that would appear to be counter-productive from a superficial perspective increase the deflection decreasing effect of turgor pressure.

## Introduction

Plants in the natural environment require adequate amounts of light for photosynthesis. Consequently, it is advantageous for them to grow tall and large in the vertical direction and efficiently extend their branches and leaves in the horizontal direction. Because they do so via independent processes while adapting to their growing environment, plants display a very diverse range of spatial forms in their natural environments.

Notwithstanding this diversity of form, there is a scaling law that is consistently present across various types of plants. This ubiquitous law may be ascribed to plants’ common goal of “efficiently resisting gravity”^1^. Greenhill derived a theoretical expression for the greatest height that a tree can grow to without buckling under its own weight, finding that this greatest height is proportional to the 2/3 power of the tree’s radius^2^. The validity of this theoretical relationship has been verified by McMahon, and it has been confirmed that it applies very accurately to trees with apparently diverse shapes^3,4^. Consequently, this scaling law has been widely used in forest science and ecology because of its simplicity and applicability^5–10^.

In addition to the mechanical constraint that the greatest height of a tree is constrained by self-weight buckling, other factors including hydraulic conditions^11,12^, wind forces^13,14^, and genetic factors^15^ have also been hypothesized as agents that determine a tree’s greatest height. The scaling law derived by Greenhill has been confirmed to be applicable only to tall and hard woody plants^16–19^, whereas the greatest height of short and soft herbaceous plants is determined, at least in part, by factors other than self-weight buckling.

From a structural mechanics perspective, fundamentally different mechanisms support the bodies of tall, woody plants and soft, herbaceous plants, respectively. Almost all of the tall, woody plant species that have been confirmed to be compatible with Greenhill’s power law have hard bodies^9,16,20,21^. The rigidity imbued by their hard bodies allows these species to resist the effects of gravity and wind. In contrast, the fleshy components of herbaceous plants are much softer than those of woody plants, having almost hollow cross-sections and extremely small diameters^16,22–25^. For herbaceous plants with such characteristics, the simple acquisition of bending rigidity is insufficient to support their bodies.

Consequently, herbaceous plants utilize the turgor pressure associated with their internal water content to maintain their rigidity^22,25–34^. In herbaceous plants, when the inflow of water exceeds the outflow of water from inside a cell, turgor pressure is generated to equalize the pressure inside and outside the cell. However, when a loss of water occurs as a result of transpiration caused by, for example, stem and branch cutting, or drying, the bending rigidity and the tension force both decrease owing to cross-sectional shrinkage^33^ and turgor pressure, respectively; a large deflection occurs instantly^34^. This is thought to be caused not only by a decrease in the bending rigidity of the plant itself because of its drying but also by the loss of the plant’s “geometric rigidity”, which is maintained by the tension forces associated with turgor pressure.

Geometric rigidity results from in-plane loading on out-of-plane rigidity^35–39^. Associated phenomena are observed in familiar situations, such as the change in pitch of a stringed musical instrument as the tension in a string is varied^37–39^. In plants, the axial tension force generated by turgor pressure creates geometric rigidity. The plants’ overall rigidity is enhanced by the geometrical rigidity, thus enabling plants to support their stems, branches, and leaves, notwithstanding their soft and thin characteristics. However, we should note that the geometric rigidity aids flexural rigidity, but both are different because the flexural rigidity can be attributed to the elastic modulus and moment of inertia, and the geometric rigidity is generated by the tension force.

The scaling law derived by Greenhill does not consider the effect of geometric rigidity, and is based on the assumption that the substances of which plants are composed possess adequate material rigidity to sustain the plants’ conformations. The majority of studies of scaling laws in forest science and botany have failed to incorporate the role of geometric rigidity; the majority of such studies apply the scaling law derived by Greenhill and dismiss the aforementioned mechanical constraints^17,18,40,41^.

An analysis of the rigidity control mechanism that utilizes the tension forces associated with turgor pressure, undertaken from a theoretical mechanics perspective, would clarify the divergence of scaling laws as they apply to woody and herbaceous plants, respectively. In addition, a theoretical description of the physical characteristics of plant deformation, with particular consideration of geometric rigidity, would be expected to have applications in the field of plant morphology; for example, in establishing methods for the nondestructive measurement of turgor pressure and Young’s modulus in plants.

The purpose of the present study was to clarify the rigidity control mechanism associated with turgor pressure and its mechanical effects in herbaceous plants, from the viewpoint of structural mechanics. We examined the geometric rigidity of the tension caused by turgor pressure by modeling branches and petioles as horizontal cantilevers subjected to self-weight and axial tension. In this model, a fourth-order governing differential equation for the plant’s deflection was derived by considering the equilibrium of the forces, and the general solution of the equation was obtained. By applying boundary conditions to the general solution, we derived an expression for the deflection of the cantilever that considers both self-weight and tension. By comparing and analyzing this equation with the comparative equation for a cantilever subjected only to its self-weight, the contribution of axial tension in decreasing a plant’s deflection was clarified.

## Methods

In this section, the mechanical characteristics of the rigidity control mechanism associated with turgor pressure in herbaceous plants are clarified by modeling a plant as a cantilever subjected to its self-weight and to an axial tension force generated by turgor pressure. The associated deflection equation is derived theoretically.

### Governing equation

**Figure 1:**
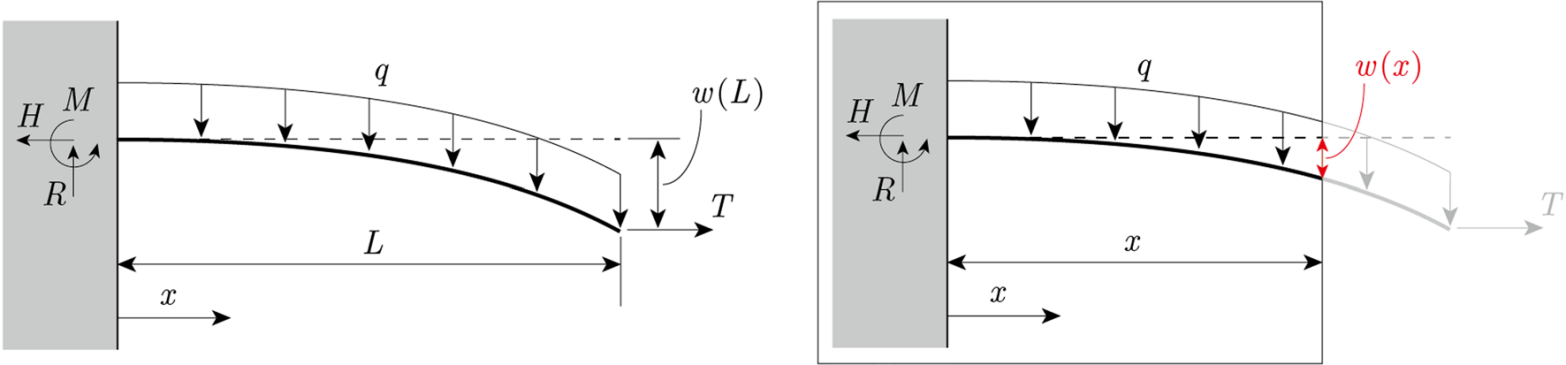
Calculation model. $L$ [m] is the length of the cantilever, $q$[N/m] is the applied self-weight, and $T$ [N] is the tension force. Based on these forces, the reaction forces $H$, $R,$ and $M$ (Eqs. 1, 2, and 3) act on the fixed end.

The calculation model is a cantilever that is subjected to its self-weight $q$ [N/m] and to a horizontal tension force $T$ [N] generated by turgor pressure (Fig. 1). The coordinate system is defined with $x=0$ at the fixed end and $x=L$ at the free end, and the bending rigidity $EI$ [N･m^2^] is assumed to be constant in the axial direction. We note that the plant model is considered a horizontally oriented thin-walled cylinder with closed ends enclosing a cavity filled with water. The specific relationship between the tension $T$ and the turgor pressure $p$ [N/m^2^] will be described later in “Relationship between tension force $T$ and turgor pressure $p$.”

When the tension force acting on a cantilever causes deflection by its own weight (Fig. 1a), assuming that the directions of action of the self-weight loading and the tension forces do not change after deformation, the reaction forces at the fixed end of the cantilever are obtained from the equilibrium of the forces, as follows:

1 $$\begin{array}{*{20}c} {H = T, } \\ \end{array}$$

2 $$\begin{array}{*{20}c} {R = qL,} \\ \end{array}$$

3 $$\begin{array}{*{20}c} {M = \frac{{qL^{2} }}{2} - Tw\left( L \right).} \\ \end{array}$$

Using the above reaction force, the bending moment $M\left( x \right)$ associated with the choice of origin at the fixed end of the cantilever is obtained as follows (Fig. 1b):

4 $$\begin{array}{*{20}c} {M\left( x \right) = - \frac{q}{2}\left( {L - x} \right)^{2} + T\left( {w\left( L \right) - w\left( x \right)} \right).} \\ \end{array}$$

By substituting Eq. (4) into the second-order differential equation describing the beam, the following second-order equation is obtained:

5 $$\begin{array}{*{20}c} { - EI\frac{{d^{2} w}}{{dx^{2} }} = - \frac{q}{2}\left( {L - x} \right)^{2} + T\left( {w\left( l \right) - w\left( x \right)} \right).} \\ \end{array}$$

The second-order derivative with respect to $x$ in the above equation yields the following final governing equation:

6 $$\begin{array}{*{20}c} {\frac{{d^{4} w}}{{dx^{4} }} - k^{2} \frac{{d^{2} w}}{{dx^{2} }} = \frac{{qk^{2} }}{T},} \\ \end{array}$$

where the parameter $k$ in Eq. (6) is given by:

7 $$\begin{array}{*{20}c} {k = \sqrt{\frac{T}{EI}} .} \\ \end{array}$$

### General solution

The following solution for *w*(*x*) was obtained from Eq. (6) using Mathematica:

8 $$\begin{array}{*{20}c} {w\left( x \right) = \frac{{e^{kx} }}{{k^{2} }}c_{1} + \frac{{e^{ - kx} }}{{k^{2} }}c_{2} + c_{3} x + c_{4} - \frac{{qx^{2} }}{2T},} \\ \end{array}$$

where ${c}_{1},{c}_{2},\dots ,{c}_{4}$ are arbitrary constants. To consider the deflection $w(L)$ at the free end $(x=L)$, we selected the following equations as the boundary conditions at the fixed end $(x=0)$:

9 $$\left\{ {\begin{array}{*{20}l} {{\text{at}} x = 0,} \hfill & {w = 0} \hfill \\ {{\text{at}} x = 0,} \hfill & {\frac{dw}{{dx}} = 0,} \hfill \\ {{\text{at}} x = 0,} \hfill & {\frac{{d^{2} w}}{{dx^{2} }} = - \frac{{k^{2} }}{T}\left( { - \frac{{ql^{2} }}{2} + Tw\left( l \right)} \right),} \hfill \\ {{\text{at}} x = 0, } \hfill & {\frac{{d^{3} w}}{{dx^{3} }} = - \frac{{k^{2} }}{T}ql. } \hfill \\ \end{array} } \right.$$

By using these boundary conditions, the final equation for the deflection $w\left(x\right)$ is derived as:

10 $$\begin{aligned} w\left( x \right) = & \frac{{qe^{ - kx} }}{{2k^{2} T\left( {e^{2kL} + 1} \right)}}\left( {2e^{kL} - 4e^{kL + kx} + 2e^{kL + 2kx} + 2kLe^{2kL} - 2kLe^{2kx} } \right. \\ & + \left. {ke^{2kL + kx} \left( {2kLx - 2L - kx^{2} } \right) - ke^{kx} \left( {kx^{2} - 2kLx - 2L} \right)} \right). \\ \end{aligned}$$

Substitution of $x=L$ into the above equation produces the equation for the maximum deflection:

11 $$\begin{array}{*{20}c} {w_{T} = \frac{q}{{k^{2} T}}\left( {1 + \frac{{k^{2} L^{2} }}{2} - {\text{sech}}kL - kL\tanh kL} \right).} \\ \end{array}$$

### Introduction of decrease rate ${D}_{R}$ associated with turgor pressure

In this study, we evaluated the effect of the tension force generated by turgor pressure on the deflection of a beam by comparing this pressure-induced deflection with the maximum deflection of a beam subjected to self-weight only. The maximum deflection ${w}_{S}$ for self-weight only is calculated using the following equation:

12 $$\begin{array}{*{20}c} {w_{S} = \frac{{qL^{4} }}{8EI}.} \\ \end{array}$$

By using parameter $k$ in Eqs. (7), (12) is transformed to the following equation:

13 $$\begin{array}{*{20}c} {w_{S} = \frac{{k^{2} qL^{4} }}{8T}.} \\ \end{array}$$

By dividing the equation expressing the maximum deflection by the tension as expressed in Eq. (11), using the deflection equation for the case without tension, the following equation is obtained:

14 $$\begin{aligned} R_{w} \left( x \right) = & \frac{{4e^{ - kx} }}{{k^{4} L^{4} \left( {e^{2kL} + 1} \right)}}\left( {2e^{kL} - 4e^{kL + kx} + 2e^{kL + 2kx} + 2kLe^{2kL} - 2kLe^{2kx} } \right. \\ & + \left. {ke^{2kL + kx} \left( {2kLx - 2L - kx^{2} } \right) - ke^{kx} \left( {kx^{2} - 2kLx - 2L} \right)} \right). \\ \end{aligned}$$

We now introduce the relative coordinate ${R}_{x}$, given by:

15 $$\begin{array}{*{20}c} {R_{x} = \frac{x}{L}.} \\ \end{array}$$

By substituting Eq. (15) into Eq. (14), the following equation expressing the relative deflection ${R}_{w}({R}_{x})$ in the relative coordinate system is obtained:

16 $$\begin{aligned} R_{w} \left( {R_{x} } \right) = & \frac{{4e^{{ - kLR_{x} }} }}{{k^{4} L^{4} \left( {e^{2kL} + 1} \right)}}\left( {2e^{kL} - 4e^{{kL\left( {1 + R_{x} } \right)}} + 2e^{{kL\left( {1 + 2R_{x} } \right)}} + 2kLe^{2kL} - 2kLe^{{2kLR_{x} }} } \right. \\ & + \left. {e^{{kL\left( {2 + R_{x} } \right)}} \left( {2k^{2} L^{2} R_{x} - 2kL - k^{2} L^{2} R_{x}^{2} } \right) - e^{{kLR_{x} }} \left( {k^{2} L^{2} R_{x}^{2} - 2k^{2} L^{2} R_{x} - 2kL} \right)} \right). \\ \end{aligned}$$

We introduce the dimensionless parameter $K$ as follows:

17 $$\begin{array}{*{20}c} {K = kL.} \\ \end{array}$$

Applying this definition of $K$ in Eq. (16), the final equation for the relative deflection is obtained as:

18 $$\begin{aligned} R_{w} \left( {R_{x} } \right) = & \frac{{4e^{{ - KR_{x} }} }}{{K^{4} \left( {e^{2K} + 1} \right)}}\left( {2e^{K} - 4e^{{K\left( {1 + R_{x} } \right)}} + 2e^{{K\left( {1 + 2R_{x} } \right)}} + 2Ke^{2K} - 2Ke^{{2KR_{x} }} } \right. \\ & + \left. {e^{{K\left( {2 + R_{x} } \right)}} \left( {2K^{2} R_{x} - 2K - K^{2} R_{x}^{2} } \right) - e^{{KR_{x} }} \left( {K^{2} R_{x}^{2} - 2K^{2} R_{x} - 2K} \right)} \right). \\ \end{aligned}$$

Replacing ${R}_{x}=1$ in Eq. (16), the maximum deflection ratio ${R}_{{w}_{max}}$ , which represents the ratio of the two maximum deflections, is given by:

19 $$\begin{array}{*{20}c} {R_{{w_{max} }} = \frac{4}{{K^{4} }}\left( {2 + K^{2} - 2{\text{sech}}K - 2K\tanh K} \right).} \\ \end{array}$$

By using this maximum deflection ratio, the rate of decrease in the maximum deflection, ${D}_{R}$ [%], is defined as follows:

20 $$\begin{array}{*{20}c} {D_{R} = \left( {1 - R_{{w_{max} }} } \right) \times 100.} \\ \end{array}$$

For example, when the rate ${D}_{R}=20\mathrm{\%}$ (as calculated using Eq. 20), it means that the maximum deflection including tension is 20% smaller than the maximum deflection under self-weight only, and that the ratio of the two deflections ${R}_{w}$ = 0.8 in this example.

### Derivation of the numerical relationship between $K$ and ${D}_{R}$

Equations (19) and (20) indicate how the dimensionless parameter $K$ is related to the rate of decrease in the maximum deflection ${D}_{R}$. To ensure the applicability of Eq. (20), it should be written explicitly in terms of $K$. However, it is extremely difficult to solve exactly for $K$. Furthermore, if we consider the case of deflection caused only by self-weight (with no contribution from the tension force), the dimensionless parameter $K$ is necessarily zero, and the rate of decrease of deflection, ${D}_{R}$, should also be zero. However, when $K=0$, Eq. (18) becomes singular. This occurs because the present theory assumes the existence of a tension force.

Consequently, in this study, the value of $K$ that will validate Eq. (20) is discretely obtained using the secant method, and interpolated using a regression model that satisfies the constraint that ${D}_{R}=0$ when $K=0$. This allows the derivation of an easily usable expression for the relationship between the decrease rate ${D}_{R}$ and the parameter $K$.

First, Eq. (16) can be transformed to the following form:

21 $$\begin{array}{*{20}c} {\left( {100 - \frac{800}{{K^{4} }}\left( {1 + \frac{{K^{2} }}{2} - {\text{sech}}K - K\tanh K} \right)} \right) - D_{R} = f\left( {K,D_{R} } \right).} \\ \end{array}$$

For any discrete value of the rate ${D}_{R}$ = 1, 2, …, 99 [%], the corresponding value of the parameter $K$ may be calculated by using Eq. (21). In this study, the value of $K$ is changed in increments of $\Delta K$ to search for the particular interval $\left[{K}_{0},{K}_{1}\right]$ inside which $f\left( {K_{0} ,D_{R} } \right) \cdot f\left( {K_{1} ,D_{R} } \right) < 0$. Using ${K}_{0}$ and ${K}_{1}$ as the initial values, iterative calculations are performed using the following equations:

22 $$K_{{\left( {m + 1} \right)}} = K_{\left( m \right)} - f\left( {K_{\left( m \right)} ,D_{R} } \right)\frac{{K_{\left( m \right)} - K_{{\left( {m - 1} \right)}} }}{{f\left( {K_{\left( m \right)} ,D_{R} } \right) - f\left( {K_{{\left( {m - 1} \right)}} ,D_{R} } \right)}}\quad \left( {m = 1,{ }2, \ldots } \right).$$

We use the following equation as a criterion to test for convergence:

23 $$\begin{array}{*{20}c} {\left| {\frac{{K_{\left( m \right)} - K_{{\left( {m - 1} \right)}} }}{{K_{\left( m \right)} }}} \right| \le \delta = 1.0 \times 10^{ - 5} .} \\ \end{array}$$

### Relationship between tension force $T$ and turgor pressure $p$

**Figure 2:**
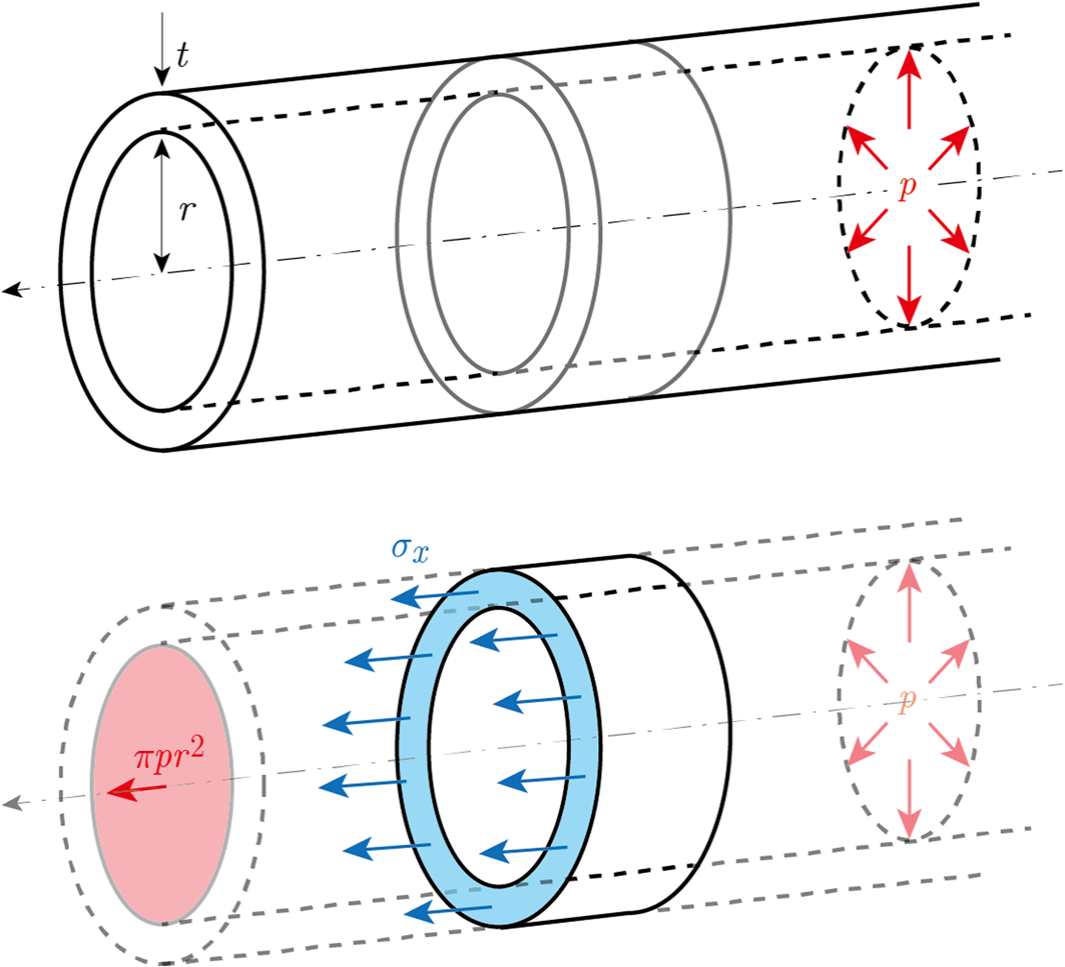
Creation of tension force owing to turgor pressure. In the thin hollow cylinder with an inner radius $r$ [m] and thickness $t$ [m], the tension force $T=\pi p{r}^{2}$ [N] (Eq. 25) acts owing to the turgor pressure $p [\mathrm{N}/{\mathrm{m}}^{2}]$.

In this section, we describe our model of a simple form (such as single-celled plant) as a thin-walled cylinder with closed ends, enclosing a cavity filled with water. We derive an equation relating the turgor pressure $p$ to the tension $T$. Suppose that turgor pressure $p$ is generated inside a hollow cylindrical structure of inner radius $r$ and epidermal thickness $t$, as shown in Fig. 2.

In this case, the force acting on a section of the cylinder is balanced by the sum of the pressures on the upper and lower surfaces of the section, resulting in an axial stress ${\sigma }_{x}$ expressed as follows:

24 $$\begin{array}{*{20}c} {\sigma_{x} = \frac{pr}{{2t}}.} \\ \end{array}$$

Since Eq. (24) expresses a force per unit area, multiplying the equation by the appropriate area produces an expression for the relationship between turgor pressure $p$ and tension $T$:

25 $$\begin{array}{*{20}c} {T = 2\sigma_{x} \pi rt = \pi pr^{2} .} \\ \end{array}$$

Moreover, the moment of inertia $I$ of a thin-walled cylinder is given by

26 $$\begin{array}{*{20}c} {I = \pi \left( {r + \frac{t}{2}} \right)^{3} t.} \\ \end{array}$$

The dimensionless parameter $K$ may be expressed in terms of turgor pressure $p$, inside diameter $r$, and epidermal thickness $t$, as follows:

27 $$\begin{array}{*{20}c} {K = \sqrt {\frac{{pr^{2} }}{{E\left( {r + \frac{t}{2}} \right)^{3} t}}} L. } \\ \end{array}$$

If we assume that the epidermal thickness $t$ is extremely small and that higher-order terms in $t$ can be neglected, the parameter $K$ may finally be expressed as follows:

28 $$\begin{array}{*{20}c} {K = \sqrt {\frac{{pL^{2} }}{Ert}.} } \\ \end{array}$$

Furthermore, we introduce the dimensionless quantities ${\lambda }_{t}$ for the epidermal thickness and ${\lambda }_{s}$ for the slenderness as follows:

29 $$\begin{array}{*{20}c} {\lambda_{t} = \frac{r}{t}, \lambda_{s} = \frac{L}{r}.} \\ \end{array}$$

By using Eq. (29), Eq. 28) can be rewritten as:

30 $$\begin{array}{*{20}c} {K = \sqrt {\frac{p}{E}\lambda_{s}^{2} \lambda_{t} } } \\ \end{array}$$

## Results and discussion

### Effect of parameter $K$ on decrease in deflection

**Figure 3:**
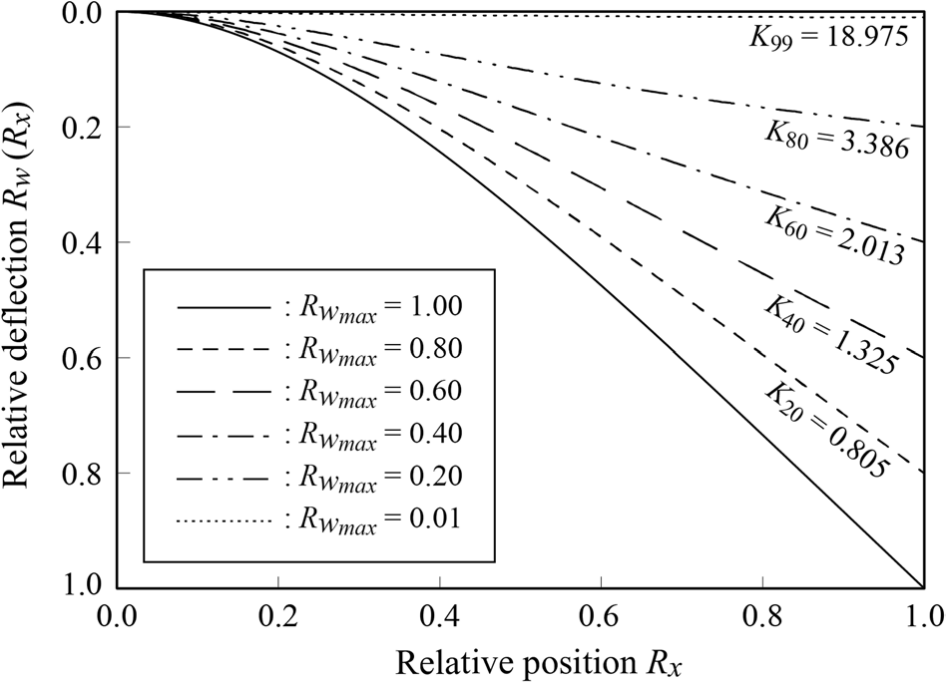
Relative deflection curve. ${R}_{x}$(Eq. 15) is a relative coordinate at the origin as a fixed end, and ${R}_{w}$ (Eq. 18) is the ratio of the deflection including the tension force to the maximum deflection with only self-weight. $R_{{w_{max} }}$ (Eq. 19) indicates its maximum value (at $x=L$). If this value is smaller, it implies that the deflection is suppressed. The value of $K$ corresponding to each deflection curve is shown in the figure.

In Fig. 3, we display the relative deflection curve when tension is considered, using the maximum deflection at the free end corresponding to consideration of self-weight only. The horizontal axis represents the relative coordinate ${R}_{x}$ in Eq. (15), and the vertical axis represents the dimensionless deflection ${R}_{w}({R}_{x})$ in Eq. (16). The values of ${K}_{{D}_{R}}$ corresponding to each $R_{w_{max}}$ are also shown in the figure.

As illustrated in the figure, the effect of decreasing the relative maximum deflection ${R}_{w_{max}}$ increases with increasing parameter $K$.

For small values of $K$, the deflection curve exhibits a curvilinear shape with respect to the relative position ${R}_{x}$. However, as $K$ increases, the decrease in the deflection increases; when ${R}_{w_{max}}=0.20$ (corresponding to a rate of decrease in deflection ${D}_{R}=80\mathrm{\%}$), the deflection shape is almost linear.

Moreover, Eq. (30) indicates that in addition to simply increasing the turgor pressure $p$, the parameter $K$ may also be increased by selecting a softer material with a smaller elastic modulus and a more elongated and thinner shape. This suggests that the ability of herbaceous plants, which are characterized by long, soft, and thin walls, to support their own bodies does not occur *notwithstanding* their softness and slenderness, but rather because of their softness and slenderness.

Although the model used in this study is a horizontal cantilever, it is expected that tension would similarly decrease the deflection in a vertically elongated model. This suggests the need to reconsider the mechanical constraints that have regularly been dismissed in studies of plant-scaling laws. However, this calculation model is only appropriate for horizontal petiole and is not adequate to consider the vertical stem. For the vertical stem, the self-buckling formulation is needed because buckling can occur owing to self-weight.

### Relationship between parameter $K$ and rate of decrease ${D}_{R}$

**Figure 4:**
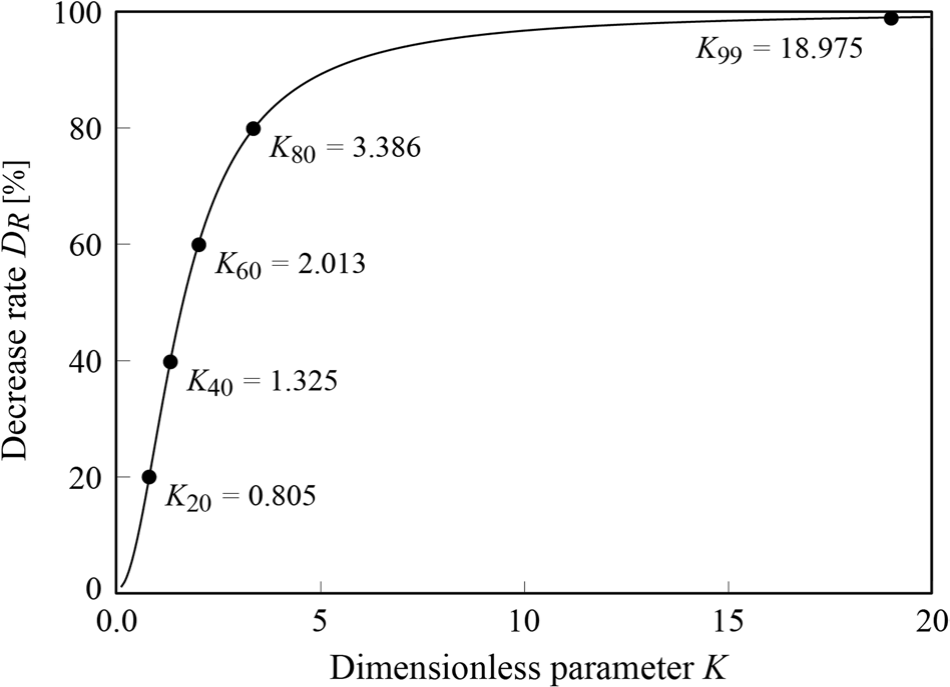
Relationship between the parameter $K$ (Eqs. 17 and 30) and decrease rate ${D}_{R}$ (Eq. 20). The larger the dimensionless parameter $K$, the greater the decrease rate ${D}_{R}$, and the self-weight deflection is suppressed. $K$ increases with a decrease in the elastic modulus $E$ and an increase in slandering and thinning; we note that the increase in $K$ is promoted by the properties of herbaceous plants.

In Fig. 4, we show the rate of decrease of ${D}_{R}$ (as expressed in Eq. 20) in the range $0<K\le 20$. The vertical axis represents the rate of decrease in the deflection ${D}_{R}$, and the horizontal axis represents the dimensionless parameter $K$.

For values of the dimensionless parameter in the range $0<K\le 3$, the rate of decrease of ${D}_{R}$ exhibits a strong, positive linear dependence on the value of $K$. However, for values of $K>3$, the strength of this dependence diminishes and deviates from linearity. As $K$ increases towards infinity, the rate of decrease of ${D}_{R}$ asymptotically approaches 100%. The rate of decrease of ${D}_{R}$ reaches 99% at a value of ${K}_{99}=18.795$. For values of ${K}_{99}\ge 18.795$, more than 99% of the deflection that would occur in the case where only self-weight acts, may be eliminated.

The absence of turgor pressure implies that the deflection occurs only under the influence of self-weight. Therefore, a rate of decrease of deflection ${D}_{R}=0$ is expected for $K=0$. However, as shown in Eq. (19), this boundary condition cannot be satisfied, because the maximum deflection ratio is undefined when $K=0$. Therefore, we use regression analysis to derive a simple relationship between ${D}_{R}$ and $K$ that satisfies the aforementioned boundary condition.

### Derivation of simple equation by regression analysis on $K$*-*${D}_{R}$ relation

**Figure 5:**
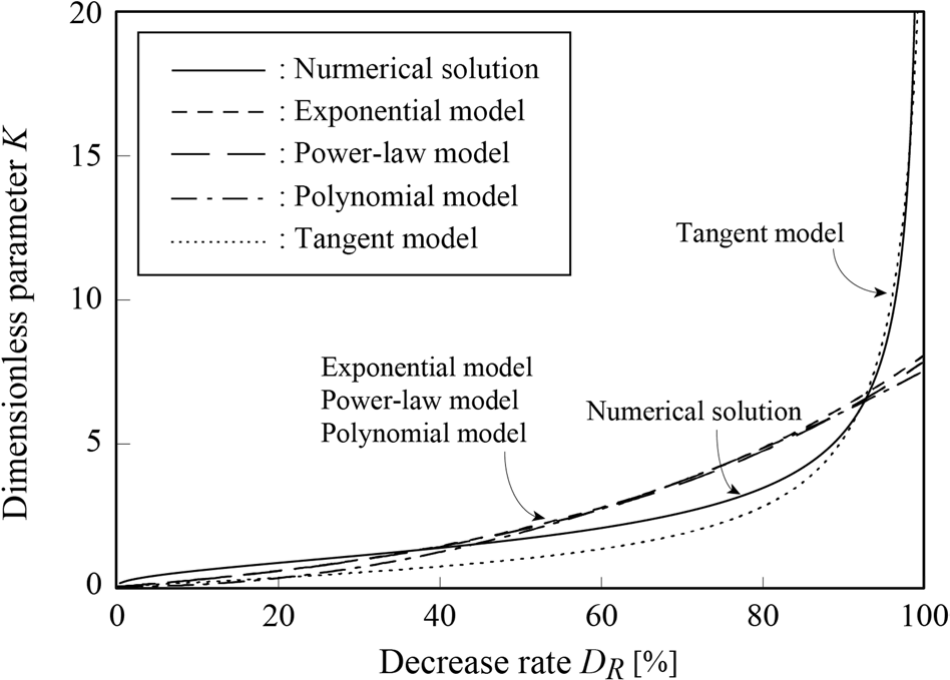
Relationship between the decrease rate ${D}_{R}$ and parameter $K$, and the regression model (Eqs. 31–34). ${D}_{R}$ increases with $K$.

In Fig. 5, we show the relationship between the rate of decrease ${D}_{R}$ [%] and the dimensionless parameter $K$, using the solution of Eq. (18) and the method described in the previous section. The horizontal axis represents the rate of decrease in deflection ${D}_{R}$ and the vertical axis represents the dimensionless parameter $K$.

The dimensionless parameter $K$ displays a linear relation with respect to the rate of decrease ${D}_{R}$, with a low gradient for the rate of decrease in the range $0<{D}_{R}\le 60\mathrm{\%}$. In contrast, for ${D}_{R}\ge 60\mathrm{\%}$, the relation becomes curvilinear and diverges to infinity as ${D}_{R}$ approaches 100%. The curves drawn with non- solid lines are regression curves obtained by regression analysis, using the following regression models to derive a simple relationship between $K$ and ${D}_{R}$:

31 $${\text{Exponential}}\,{\text{model:}} \,K = e^{{P_{1} D_{R} }} - 1$$

32 $${\text{Power - law}}\,{\text{ model:}} \,K = P_{2}^{{D_{R} }} - 1$$

33 $${\text{Polynomial}}\,{\text{ model:}} \,K = P_{3} D_{R}^{2}$$

34 $${\text{Tangent }}\,{\text{model:}} \,K = \tan P_{4} D_{R}$$

where ${P}_{1} \sim {P}_{4}$ are regression coefficients. All the above regression models satisfy the condition that $K=0$ when ${D}_{R}=0$ and include only one regression parameter, making them extremely simple models. Detailed results of the nonlinear regression analysis in are listed in Table 1.

**Table 1:** Result of regression analysis.

Model	Exponential	Power-law	Polynomial	Tangent
Parameter	${P}_{1}$	${P}_{2}$	${P}_{3}$	${P}_{4}$
Estimated value	2.221 × 10^–2^	1.022	7.569 × 10^–4^	1.530 × 10^–2^
*p* value	2.0 × 10^–16^	2.0 × 10^–16^	2.0 × 10^–16^	2.0 × 10^–16^
AIC	362.85	362.85	372.19	179.82

As shown in Table 1, all parameters are significant because the p-values for all models are below the significance level of $\alpha =0.05$. However, as can be observed from the graphs, the errors in the numerical solution of Eq. (18) are not small for all the models. In particular, the three models other than the tangent model display significant errors when ${D}_{R}\ge 50\mathrm{\%}$. The values of the Akaike information criterion (AIC) for these models are almost the same; only the tangent model has a relatively small value of the AIC.

The above-mentioned results indicate that the tangent model is the most appropriate model for expressing the relationship between $K$ and ${D}_{R}$ among the regression models shown in Eqs. (31–34). However, it should be noted that although the tangent model represents the relation very accurately when ${D}_{R}\ge 90\mathrm{\%}$, it underestimates the value of the parameter $K$ in the range where $0\le {D}_{R}\le 90\mathrm{\%}$.

## Conclusions

In this study, the branches and petioles of herbaceous plants were modeled as horizontal cantilevers subjected to both self-weight and axial tension generated by turgor pressure, and a deflection equation was theoretically derived by considering the effect of geometric rigidity caused by the tension generated by turgor pressure. By comparing and discussing this equation with the deflection equation for a cantilever subjected only to self-weight, the following conclusions were obtained:

1. The horizontal tension created by turgor pressure significantly decreased the deflection owing to self-weight. The deflection-decreasing effect of the tension force could be expressed using four parameters: Young’s modulus, turgor pressure, a dimensionless parameter related to the slenderness, and a dimensionless parameter related to the epidermal thickness of the plant.
2. The deflection-decreasing effect owing to the tension force was greater for softer materials with a smaller Young’s modulus E. The more slender the cylinder, the greater was the deflection-decreasing effect due to tension. This finding was consistent with the characteristics of herbaceous plants^16,23,42^, and it could be inferred that herbaceous plants support themselves through the tension generated by turgor pressure.
3. The model used in this study was a horizontally elongated cantilever, but a similar deflection-decreasing effect may be expected in the case of a vertically elongated trunk or stem. The results obtained in this study suggest the that the mechanical constraints that have been dismissed in previous scaling law studies need to be reconsidered in future work.

In the future, we intend to study the applicability of the formula derived in this study based on the structural mechanics theory to the nondestructive estimation of turgor pressure and Young’s modulus in real plants, using stocked statistical data in the field of botany and related experimental approaches. In addition, by applying the findings and formulation methods obtained in this study, we will formulate an expression for calculating the greatest height of herbaceous plants from the viewpoint of mechanics theory, taking geometric rigidity into consideration, with the purpose of obtaining an understanding of scaling laws obtaining in all plants, including soft herbaceous plants. Moreover, to ensure its sufficient applicability to multicellular plants^43^, we seek to clarify the effects of the interactions in multicellular plants on the tension force.

## Data Availability

The datasets generated during and/or analysed during the current study are available from the corresponding author on reasonable request.
